# A Rare Presentation of a Neuroendocrine Tumor: A Case Report

**DOI:** 10.7759/cureus.83430

**Published:** 2025-05-03

**Authors:** Elisheva Knopf, Camryn R Marshall, Leah Leidy, Parvathi Perumareddi

**Affiliations:** 1 Department of Medicine, Florida Atlantic University Charles E. Schmidt College of Medicine, Boca Raton, USA; 2 Department of Family Medicine, Florida Atlantic University Charles E. Schmidt College of Medicine, Boca Raton, USA

**Keywords:** disease prevention, hepatic metastasis, hypercalcemia of malignancy, neuroendocrine tumor, primary care

## Abstract

Neuroendocrine tumors (NETs) are slow-growing, rare malignancies that originate from neuroendocrine cells and may be classified as functional, secreting hormones that produce clinical symptoms, or non-functional, often remaining asymptomatic until advanced stages. Primary gastrointestinal NETs in the esophagus (E-NETs) are rare, accounting for only 0.4%-2% of esophageal malignancies. Due to their rarity, often subtle early symptoms, and being mostly non-functional tumors, they are frequently diagnosed at an advanced stage with metastasis. This case report describes a patient who presented to the emergency department (ED) with a three-week history of weakness, hypophonia, and dysphonia, two months after carpal tunnel surgery. The patient had a past medical history (PMH) significant for hypertension and hypothyroidism and had no recent symptoms or medical concerns upon presenting for her preoperative examination for carpal tunnel surgery a few months prior. Before this preoperative appointment, she had an established primary care physician (PCP); however, she never had regular follow-ups or consistent monitoring. The patient’s initial workup in the ED revealed hypercalcemia and elevated liver enzyme levels. In addition to these abnormal lab results, a subsequent right upper quadrant ultrasound showed multiple lesions in the liver, indicative of metastasis. The next day, the patient had an abdominal CT scan performed, which revealed wall thickening in the distal esophagus, suggestive of neoplasm or malignancy. The tumor was thought to have originated at the gastroesophageal junction and metastasized to the liver. The patient had a poor prognosis and passed away from the metastatic tumor within two weeks of diagnosis. This case underscores the aggressive nature and atypical presentations of E-NETs, including rare paraneoplastic syndromes, such as hypercalcemia. It also underscores that serious malignancies can develop in patients without traditional risk factors. While standardized guidelines for E-NET follow-up and management remain limited, this case reinforces the importance of maintaining a high index of suspicion for atypical symptoms and ensuring adherence to routine preventive care, which may facilitate earlier detection of rare but aggressive tumors.

## Introduction

Neuroendocrine tumors (NETs), formerly known as carcinoid tumors, originate from neuroendocrine cells and exhibit unique characteristics of both nerve and endocrine cells. Neuroendocrine cells are distributed throughout various tissues, and when they transform to malignancy, they can lead to a wide spectrum of symptoms, depending on location and the type of hormone the tumor secretes [1]. NETs are typically slow-growing tumors and may be classified as functioning, meaning the tumor secretes hormones such as serotonin, insulin, glucagon, or gastrin, or non-functioning, where the tumor does not secrete hormones. NETs most commonly arise in the gastrointestinal tract and bronchopulmonary system [2]. Prognosis varies based on the tumor’s origin, and tumors presenting late and with metastases generally carry the poorest prognosis, while earlier detection is usually associated with a better outcome [3].

Gastrointestinal NETs can develop anywhere along the gastrointestinal tract but occur most frequently in the midgut, followed by the foregut and the hindgut [2]. Once the diagnosis is suspected, it is confirmed through immunohistochemical staining of biomarkers such as chromogranin A, synaptophysin, and neuron-specific enolase [2]. Primary gastrointestinal NETs in the esophagus are rare, largely non-functional, and account for only 0.4%-2% of esophageal malignancies [4]. The incidence of esophageal NETs (E-NET) has been rising, likely due to increased gastrointestinal screenings, such as upper gastrointestinal endoscopies, as early-stage symptoms are typically minimal [5,6]. Given their rarity, limited data exist on prognosis and optimal treatment strategies for these tumors.

## Case presentation

Our patient, a 74-year-old Caucasian female with a past medical history (PMH) of hypertension, hypothyroidism, and right-sided carpal tunnel surgical repair two months prior, presented to the Emergency Department (ED) at the recommendation of her primary care physician (PCP) for possible evaluation of Guillain-Barre Disease.

Three weeks prior to presentation at the ED, she had a postoperative examination after her carpal tunnel surgery, at which time she was found to have elevated blood pressure. She was taking Olmesartan at the time, and the dosage was increased from 20 mg to 40 mg PO daily. Her only other medication at the time was levothyroxine. Of note, she did not see her PCP for regular follow-up prior to the preoperative visit for carpal tunnel surgery two months prior. A few days later, while at an outdoor event, she experienced lightheadedness that resolved with sitting down with her head between her legs. Over the next three weeks, she noticed progressive generalized weakness in her hands and bilateral lower extremities, reduced appetite with a resultant unintentional weight loss of 11 lbs, hoarse voice, significant fatigue consisting of sleeping throughout the day and night sweats, six days of constipation, and some hand and foot intention tremor. Within the next two days, she experienced worsening lower extremity weakness and loss of ability to ambulate. This led her to seek care with her PCP, who recommended she go to the ED.

**Figure 1:**
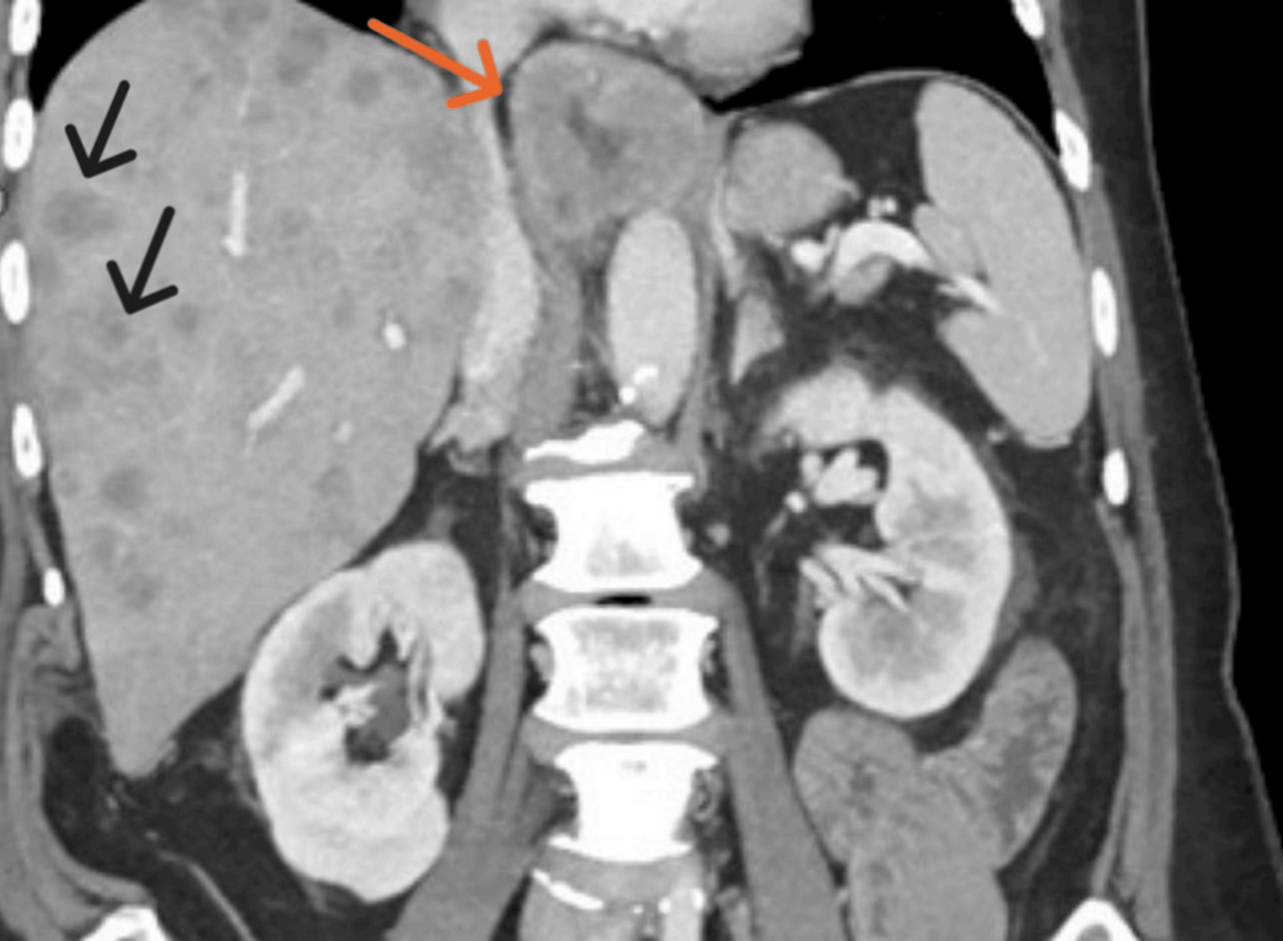
CT of the abdomen, pelvis, and chest Analysis reveals mass-like wall thickening of the distal esophagus (orange arrow), suspected of neoplasm/malignancy. Innumerable hepatic masses (black arrows), suspected of metastases. Gastrohepatic ligament lymphadenopathy, suspected of metastases (not shown). A few pulmonary micronodules up to 3 mm (not shown).

In the ED, she was normotensive and afebrile with normal heart rate and oxygen saturation. On physical examination, she appeared weak with hypophonia and dysphonia, and her bowel sounds were diminished. The rest of her examination was unremarkable, including normal sinus rhythm, normal S1 and S2, no abdominal tenderness or distention, no hepatosplenomegaly, 5/5 strength in her upper and lower extremities, 2+ reflexes in all upper and lower extremities, and full sensation to temperature and light touch. The attending physician in the ED had ordered a comprehensive blood count (CBC), basic metabolic panel (BMP), and liver function tests (LFTs) upon patient arrival, which were remarkable, specifically for elevated white count, elevated liver enzymes, and most importantly, hypercalcemia (Table 1). The corrected calcium for hypoalbuminemia was 17.5. Of note, CBC, BMP, and LFTs had been obtained several months ago, all of which were within normal limits. Her initial chest x-ray on presentation to the ED was unremarkable. Her right upper quadrant ultrasound was significant for multiple liver lesions, suggestive of metastasis. Given these findings, blood cultures, urinalysis, ionized calcium, parathyroid hormone, lactate dehydrogenase, uric acid, thyroid studies, 1,25 hydroxyvitamin D, magnesium, and phosphorus were ordered (Table 2). A CT of the abdomen and pelvis and a CT of the chest were ordered (Figure 1) and were significant for mass-like wall thickening of the distal esophagus, likely of neoplastic/malignant origin. Innumerable hepatic masses, gastrohepatic ligament lymphadenopathy, and a few pulmonary micronodules up to 3 mm were seen, all suggestive of metastasis.

**Table 1:** Patient labs upon presentation to the Emergency Department (ED) ALT: alanine transaminase; AST: aspartate transaminase

Lab Test	Patient Result	Reference Range
White Blood Cell Count (WBC)	20.4 x 10³/µL (High)	4.0–11.0 x 10³/µL
Hemoglobin (Hb)	14.9 g/dL	13.5–17.5 g/dL (M), 12.0–15.5 g/dL (F)
Platelets	394 x 10³/µL	150–450 x 10³/µL
Absolute Neutrophil Count	15.6 x 10³/µL (High)	1.5–8.0 x 10³/µL
Absolute Monocyte Count	2.7 x 10³/µL (High)	0.2–1.0 x 10³/µL
Sodium	134 mmol/L (Low)	135–145 mmol/L
Potassium	3.9 mmol/L	3.5–5.1 mmol/L
Blood Urea Nitrogen (BUN)	21 mg/dL (High)	7–20 mg/dL
Creatinine	0.7 mg/dL	0.6–1.3 mg/dL
Alkaline Phosphatase	427 U/L (High)	44–147 U/L
AST	109 U/L (High)	8–40 U/L
ALT	202 U/L (High)	7–56 U/L
Lactate Dehydrogenase	1,312 U/L (High)	140–280 U/L

**Table 2:** Patient laboratory results following ultrasound in the ED

Lab Test	Patient Result	Reference Result
Lactate Dehydrogenase (LDH)	1,312 U/L (High)	140–280 U/L
Uric Acid	7.8 mg/dL (High)	3.5–7.2 mg/dL (M), 2.6–6.0 mg/dL (F)
Ionized Calcium	1.8 mmol/L (High)	1.11–1.3 mmol/L
Parathyroid Hormone (PTH)	2 pg/mL (Low)	10–65 pg/mL
Magnesium	1.3 mg/dL (Low)	1.7–2.2 mg/dL
Phosphorous	1.7 mg/dL (Low)	2.5–4.5 mg/dL
Thyroid Stimulating Hormone (TSH)	0.144 µIU/mL (Low)	0.4–4.5 µIU/mL
Total T3	1.4 ng/mL	0.8–2.0 ng/mL
Free T4	0.5 ng/dL (Low)	0.7–1.8 ng/dL
Parathyroid Hormone-Related Peptide (PTHrP)	8.4 pmol/L (High)	<2.0 pmol/L
Urinalysis	Within normal limits	Normal findings
Blood Culture	No growth at 72 hours	No growth

**Figure 2:**
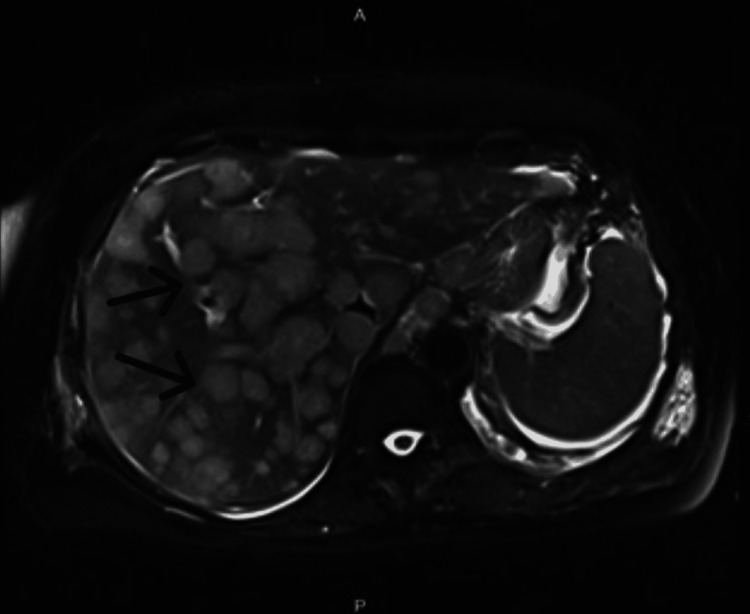
MRI of the abdomen Analysis reveals primary mass at the gastroesophageal junction (not shown) and also extensive metastatic disease to the liver (black arrows) with slight early ascites (not shown).

The patient was admitted to the Inpatient Medicine Department, at which time hematology, oncology, and endocrinology were consulted for further evaluation and management. Interventional gastroenterology was consulted for endoscopic ultrasound and biopsy of lesions to identify the primary source of the cancer and obtain a histologic description for further oncologic management. Endoscopy revealed a metastatic gastroesophageal junction (GEJ) mass with extensive hepatic metastases and gastrohepatic lymphadenopathy, but no evidence of active gastrointestinal bleeding or esophageal obstruction. Her endoscopic ultrasound (EUS) stage was uT3T2M1. Malignant infiltration into the lamina propria was noted at the GEJ. Forceps biopsy and EUS core biopsy were performed and revealed the diagnosis of an esophageal neuroendocrine tumor. MRI of the abdomen showed both the primary mass at the GEJ but also extensive metastatic disease to the liver with slight early ascites (Figure 2). The patient had a poor prognosis and passed away from her metastatic disease within two weeks of diagnosis. Of note, the patient had no family history of cancer, endocrine conditions, or neuroendocrine tumors. She also lacked any traditional risk factors for esophageal cancers, including smoking, heavy drinking, obesity, Barrett’s esophagus, trauma or accidents to the chest, or cancer/history of radiation to the chest [5].

## Discussion

This case presentation highlights a rare presentation of metastatic E-NET in a patient who exhibited minimal symptoms prior to her ED visit. E-NETs are classified by differentiation and grade, with well-differentiated NETs being exceedingly rare in the esophagus, whereas poorly differentiated neuroendocrine carcinomas (NECs) are more common and typically high-grade and aggressive. The most frequently reported symptoms of E-NETs include dysphagia, weight loss, retrosternal and epigastric pain, dysphonia, coughing, and hormone-related symptoms. Dysphagia is the most common presenting symptom, while incidental detection is rare [7].

The patient described in this case presented with dysphonia and weight loss, both consistent with typical clinical features of E-NETs. However, she also exhibited atypical symptoms, such as tingling in the extremities, potentially secondary to hypercalcemia, which is a rare paraneoplastic manifestation in a few documented cases of E-NETs [8,9]. Interestingly, despite her elevated PTHrP and associated hypercalcemia, she lacked traditional symptoms of hypercalcemia, including polyuria, kidney stones, and neuropsychiatric symptoms. Furthermore, PTHrP-mediated hypercalcemia is more commonly associated with squamous cell carcinomas or renal and breast malignancies, making this an unusual finding in the context of an E-NET [10]. The absence of overt symptoms despite significant biochemical derangement raises questions about the potential chronicity or adaptation in this patient’s case.

Although the patient had an assigned PCP, she did not engage in regular follow-up, despite having no reported barriers to care such as insurance, transportation, or health literacy challenges. This case underscores the critical importance of routine interval care and preventive health screenings. Evidence consistently shows that patients with regular PCP follow-up are more likely to receive recommended screenings, experience fewer hospitalizations, and have reduced mortality rates [11,12].

This patient had never undergone standard age-appropriate screenings, such as mammography or colonoscopy, nor did she receive routine laboratory evaluations such as CBC or CMP. While a pre-operative evaluation for carpal tunnel repair was performed a few months prior, with normal labs at that time, this encounter could not substitute for consistent annual monitoring. Regular follow-up might have facilitated earlier detection of her malignancy before progression to metastatic disease. We want to highlight the importance of consistent yearly monitoring, in addition to follow-up appointments for chronic conditions, which can assist in detecting other conditions, including that of this patient.

Another key aspect of this case is the absence of traditional esophageal cancer risk factors, such as smoking, heavy alcohol consumption, or obesity [5]. It serves as a reminder that serious pathologies can develop even in patients without classic risk profiles. Therefore, clinicians must maintain a broad differential diagnosis and emphasize comprehensive history-taking and physical examinations, regardless of perceived risk. Early recognition of subtle or atypical symptoms could prompt a timely diagnostic workup and potentially prevent disease progression. Given the rarity of E-NETs, standardized follow-up and management guidelines are limited. However, current recommendations suggest clinical evaluations every three months, imaging (CT, MRI, or ultrasound) every three to six months, and endoscopic surveillance annually or sooner if new symptoms arise [13]. Awareness of these protocols is essential for optimizing outcomes following diagnosis.

This case also brings to attention the potential presence of barriers to care. When asked directly about common barriers, such as insurance issues, transportation difficulties, limited health literacy, or fear of medical providers, the patient denied experiencing any of these. Nevertheless, it remains critical to acknowledge that barriers to care are a well-documented contributor to delays in diagnosis and lapses in follow-up [14]. To mitigate this, healthcare providers should take advantage of each patient encounter to reinforce the importance of regular follow-up and preventive screening, address any concerns or misconceptions, and assist with scheduling appointments. These efforts may help facilitate earlier detection and management of disease.

Although this patient presented with a rare and atypical neuroendocrine tumor, the lessons learned from her case have broad implications for medical practice. Emphasizing regular patient follow-up, monitoring, and comprehensive history-taking should be a priority amongst medical providers to promote better health outcomes for patients [15]. Physicians should educate their patients on the importance of these practices to promote overall wellness and help prevent potentially negative or devastating outcomes. Further, early education of medical students regarding preventative guidelines and thorough history and physicals can also aid in impacting future medical care.

## Conclusions

Although E-NETs are rare and treatment options remain limited, regular screenings and comprehensive evaluations by PCPs play a pivotal role in facilitating early detection and intervention. In our patient’s case, the absence of timely screening and consistent follow-up may have contributed to the progression to advanced malignancy that was detected late in the disease course. Interestingly, the patient had normal labs several months prior; however, she had not had any other traditional screening tests, such as a colonoscopy or mammogram. This underscores the need for increased awareness among clinicians and patients about the importance of routine preventive care, including age-appropriate cancer screenings, as well as establishing methodical practices for annual physical exams, which may help identify malignancies such as NETs at earlier, more treatable stages.
